# Supplementary material for: A heterogeneously integrated lithium niobate-on-silicon nitride photonic platform
Source: Nat Commun. 2023 Jun 13;14:3499. doi: 10.1038/s41467-023-39047-7 (PMC10264395; doi:10.1038/s41467-023-39047-7)
Supplement: Supplementary file 1 — Supplementary Information [file 41467_2023_39047_MOESM1_ESM.pdf]

Supplementary information for "A heterogeneously integrated lithium niobate-on-silicon nitride photonic platform"

Mikhail Churaev[1*], Rui Ning Wang[1*], Annina Riedhauser[2*], Viacheslav Snigirev[1], Terence Blésin[1], Charles Möhl[2], Miles H. Anderson[1], Anat Siddharth[1], Youri Popoff[2,3], Ute Drechsler[2], Daniele Caimi[2], Simon Hönl[2], Johann Riemensberger[1], Junqiu Liu[1], Paul Seidler[2,†], Tobias J. Kippenberg[1,‡]
[1]*Institute of Physics, Swiss Federal Institute of Technology Lausanne (EPFL), CH-1015 Lausanne, Switzerland*
[2]*IBM Research - Europe, Zurich, CH-8803 Rüschlikon, Switzerland*
[3]*Swiss Federal Institute of Technology Zurich (ETH Zürich), CH-8092 Zürich, Switzerland*
*These authors contributed equally to this work
Emails: ‡ tobias.kippenberg@epfl.ch, † pfs@zurich.ibm.com

CONTENTS

I. Fabrication details 2

II. Linewidth histograms for wafer map 2

III. Fiber-to-chip transmission calibration 3

IV. Electro-optic efficiency 3

V. Bending losses 4

VI. Electro-optic microring characterization 5

VII. Electro-optic frequency comb simulation 6

VIII. Supercontinuum and second-harmonic generation in hybrid waveguides 7

Supplementary Table 1 8

References 9

I. FABRICATION DETAILS

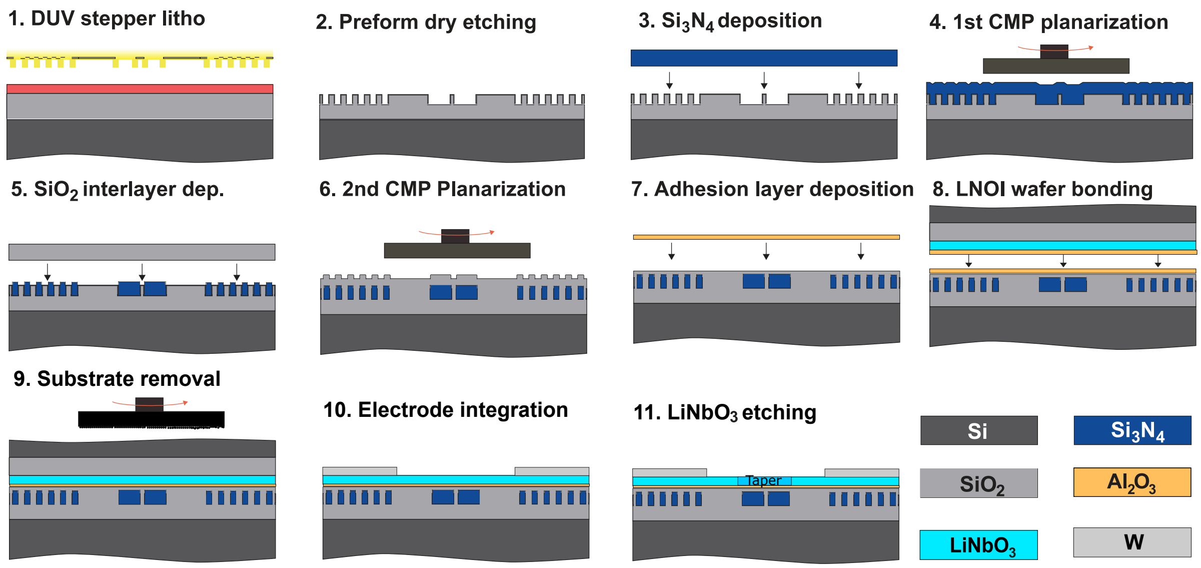

Supplementary Figure 1: Schematics of all the processing steps involved in fabrication of our heterogeneously integrated $LiNbO_3$ photonic circuits. Steps 1-5 correspond to standard Photonic Damascene process, steps 6-9 are related to surface preparation and bonding, and 10-11 are the post-bonding processing.

Fabrication steps 1-4 in figure S1 are described in detail elsewhere [1].

II. LINEWIDTH HISTOGRAMS FOR WAFER MAP

Fig S2 shows the $\kappa_0/2\pi$ histograms for the same 21 GHz FSR microring resonator, measured in 5 different fields on the bonded 2" wafer. The most probable values for each field are given in Fig 2(e) of the main manuscript.

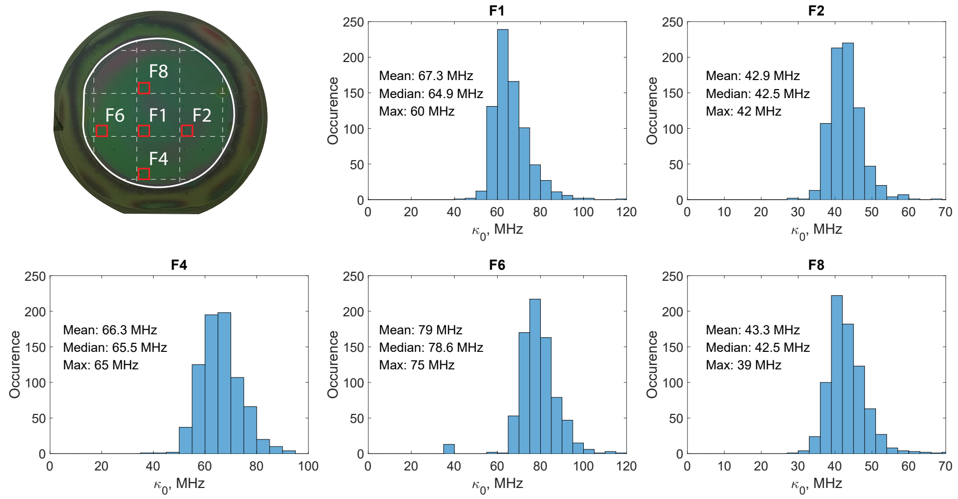

Supplementary Figure 2: Linewidth data for the wafer map presented in the main text.

III. FIBER-TO-CHIP TRANSMISSION CALIBRATION

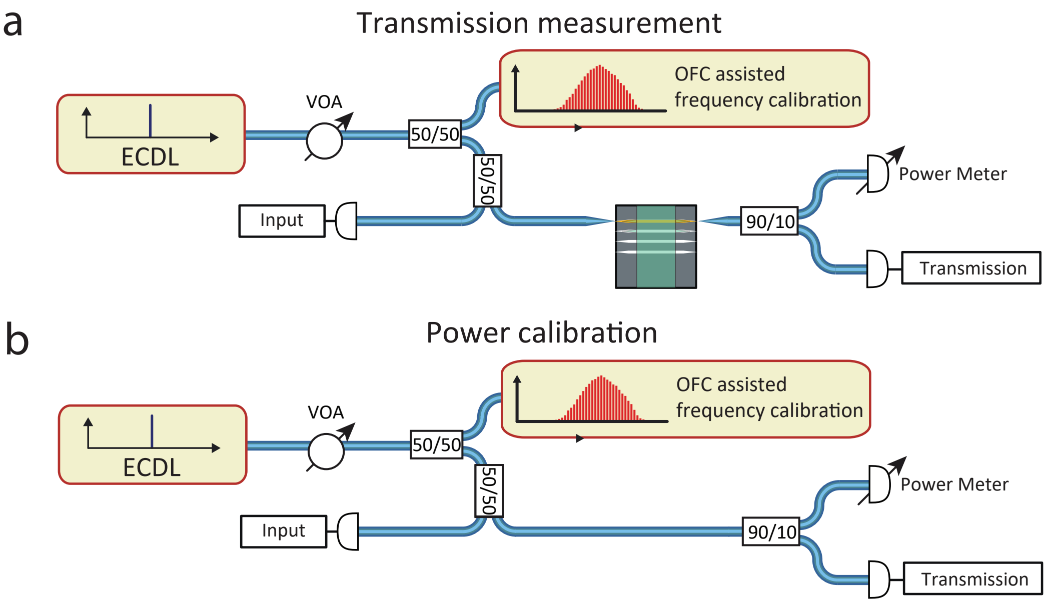

Supplementary Figure 3: Layout of fiber-to-fiber transmission measurement setup (a) and power calibration (b). ECDL - external cavity diode laser, OFC - optical frequency comb, VOA - voltage-controlled attenuator.

IV. ELECTRO-OPTIC EFFICIENCY

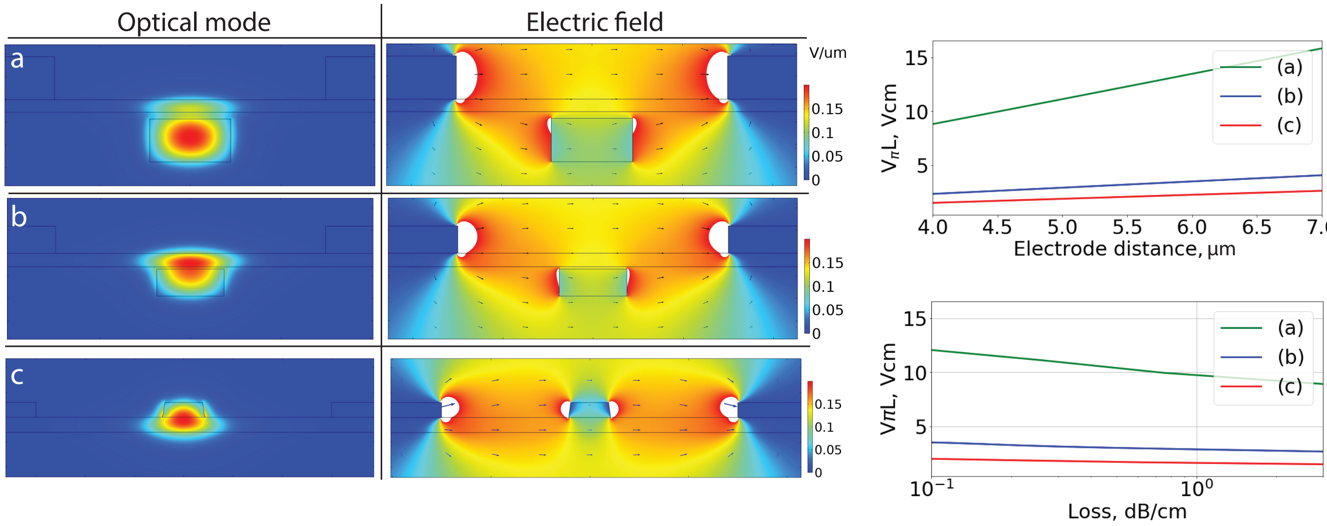

Supplementary Figure 4: Comparison of electro-optic efficiency for 3 different structures: (a) the bonded lithium niobate structure presented in this work and (b) the bonded structure with optimized geometry for electro-optic modulation. (c) typical ridge waveguide configuration. The plots on the right show simulation results for the half-wave voltage - length product ($V_\pi L$) for a MZM configuration and the corresponding accumulated linear loss due to the metal presence. White parts in electric field distribution correspond to areas with a saturation of electric field larger than 0.2 V/μm.

The main figure of merit for electro-optic modulation is the half-wave voltage - length product ($V_\pi L$) value. To

compare electro-optic efficiency for classical ridge waveguides [2, 3] and the hybrid structure presented in this work we perform FEM simulations in COMSOL Multiphysics for optical mode and electrostatic field distribution. The refractive index change under external modulation electric field E is:

$$\Delta n_{\mathrm{eo}} = \frac{1}{2} n^3 r_{33} E. \tag{1}$$

Here we consider both modulation electric field E and optical electric field E_{opt} to be coplanar with extraordinary $LiNbO_3$ crystal axis. The modulation electric field strength E is linearly proportional to the voltage applied on the electrodes so that $E = V/d_{\mathrm{eff}}$, where effective distance value d_{eff} represents just a linear scaling parameter. We calculate this parameter by simulating electrostatic and optical fields with 1V applied to the electrodes. The effective distance will be then inverse proportional to the normalized effective electric field, taking into account optical and electrostatic field overlap:

$$d_{\mathrm{eff}} = \frac{1V}{E_{\mathrm{eff}}};\ E_{\mathrm{eff}} = \frac{\int_{\mathrm{LN}} |E_{x,\mathrm{opt}}|^2 E_{x,\mathrm{el}} dS}{\int |E_{x,\mathrm{opt}}|^2 dS} \tag{2}$$

We calculate the $V_\pi L$ values for dual-arm Mach-Zehnder modulators as [4]:

$$V_\pi L = \frac{\lambda d_{\mathrm{eff}}}{2n^3 r_{33}}, \tag{3}$$

where λ is the optical wavelength.

The $V_\pi L$ value depends on the gap distance between the electrodes, so to compare the overall electro-optic efficiency we need to also take into account optical losses induced by the metal electrodes which limits the minimum electrode gap. In Figure S4 we give a comparison of half-wave voltage-length products as a function of induced optical loss for the current structure, optimized bonded structure, and classical ridge waveguide structure. As can be seen from the simulations, the bonded structure slightly compromises electro-optic efficiency ($\times 2$ factor) primarily because of weaker optical mode confinement (therefore induced mode area). As was discussed in the main text, the hybrid waveguide conceptually behaves as a ridge waveguide since in case of a ridge waveguide the electric field avoids the waveguide itself and is accumulated in the slab (see Figure S4(c) electric field distribution). Case (b) corresponds to a 1 μm wide waveguide having 600 nm thickness and 100 nm interlayer thickness bonded to a 300 nm thick lithium niobate slab. In this configuration, the mode confinement in $LiNbO_3$ reaches 40%.

V. BENDING LOSSES

We perform numerical FEM simulations to verify if the "electro-optic" configuration (600 nm thick Si_3N_4, 2 μm wide waveguides) is not dominated by whispering-gallery losses (bending losses). The high optical mode participation in lithium niobate could lead to significant radiation into the slab at waveguide turns. However, according to our simulations (cf. Figure S5(a)), the quality factors ($>10^7$) will not be dominated by bending loss for microresonators having down to 100 μm radius in this configuration. The critical point here is to minimize the silicon oxide interlayer (spacer) thickness. In the case of a thick spacer, the optical mode starts to split into high-confinement core mode and low-confinement slab mode, which leads to a large dissipation of the latter at waveguide turns (see Figure S5(b)). Unfortunately, the interlayer spacer cannot be removed fully, as it provides a flat bonding-ready surface for our hybrid structure, but for future high-participation applications, its thickness should be minimized for better performance.

To verify the model we measure Q-factors of two microresonators in the electro-optic waveguide configuration: 120 μm and 180 μm radii (Figure S5(c-d)). The spacer thickness is around 80 nm according to the cross-section SEM measurements, and waveguide width is set to be 2 μm for both resonators. The smaller ring reveals an increase in the resonance linewidth, which leads to the Q-factor of $3.9{\cdot}10^5$, while the larger ring has almost a magnitude higher Q-factor ($2.2{\cdot}10^6$), close to the ultimate measured values presented in the main manuscript for the rings having radii 225 μm and more. The latter means that for 80 nm spacer thickness the loss is not dominated by bending already at 180 μm radius. The "waving" background in panel (c) corresponds to the standing waves inside the bus waveguide and are visible due to the 16-GHz scale of the frequency axis (the total resonance linewidth in this case reaches approximately 800 MHz). We also mark these measurement results with red crosses on the chart presented in Figure S5(a).

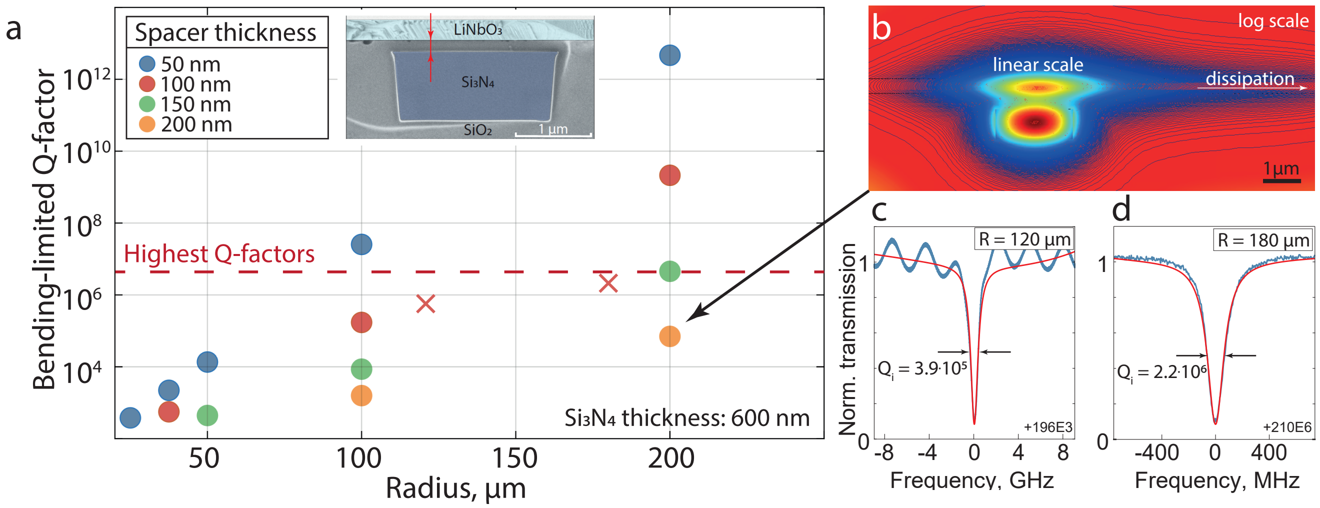

Supplementary Figure 5: Bending loss-limited Q-factors in hybrid waveguides. (a) Simulation results for the "electro-optic" waveguide geometry for different bending radii and spacer thicknesses. Crosses correspond to the measurements presented in panels (c-d). (b) Example of an optical mode dissipation in case of a thick spacer. The inner waveguide colors display linear-scale mode distribution, while the far-field lines are in log scale to visualize an optical mode dissipation in the tail, being de-coupled from the Si_3N_4 waveguide core. (c) Resonance fitting (red curve) of a 120 μm microresonator in the simulated configuration with 80 nm spacer thickness. (d) The same measurement for a resonator with 180 μm radius. Note the frequency scale change for panels (c)-(d).

VI. ELECTRO-OPTIC MICRORING CHARACTERIZATION

The linear tuning of the 20 GHz hybrid device is measured by locking the CW laser on a resonance of the microresonator via a PDH stabilization as shown in figure S6. A 30 Volts peak-to-peak ramp signal is applied on the electrodes of the device and the PDH error signal is obtained by phase-modulating the pump laser (at a frequency in the range of 500 – 800 MHz) before coupling to the cavity, using an electro-optic modulator (EOM, iXblue MPX-LN-0.1). The modulated signal is detected after the resonator and demodulated to DC using the same RF signal. After demodulation, the baseband signal is low-pass-filtered and sent to a PID servo-controller (Toptica FALC). The servo output is then measured and processed after due calibration to estimate the linear tuning coefficient of the device as 41.69 MHz/V.

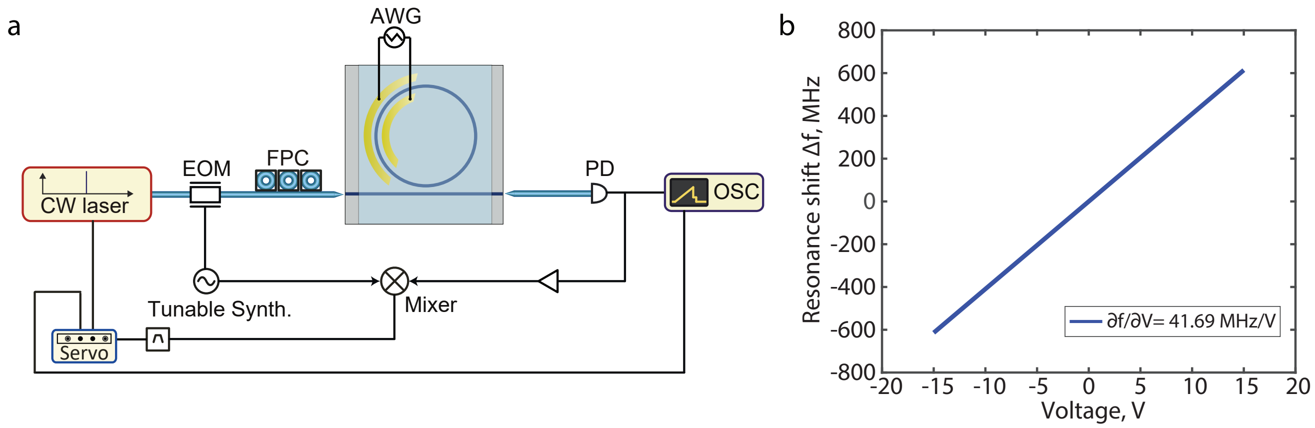

Supplementary Figure 6: Linear resonance tuning measurements. a) Experimental schematic of linear tuning measurement via Pound-Drever-Hall laser stabilization method. The CW laser is locked to the microresonator resonance and on applying an electrical signal on the electrodes, the calibrated servo output provides the linear tuning coefficient of the device under test. b) Resonance shift versus applied voltage for a 21 GHz FSR resonator. The linear tuning coefficient at DC is $\Delta f/V$ = 41.69 MHz/V.

The metal electrodes form a capacitive element. The value of the associated capacitance is an essential information to characterize the modulation efficiency of traveling-wave devices, and the single electro-optic coupling of resonant ones. Since the microwave response of $LiNbO_3$ is populated by piezoelectric features and the low frequency response is modified by the probes, the static capacitance is obtained here by taking the slope of the imaginary part of the admittance at high frequencies. The admittance is obtained from a microwave reflection measurement

$$Y_{11} = \frac{1}{Z_0} \frac{1 - S_{11}}{1 + S_{11}} \tag{4}$$

for 1-port devices, with a characteristic impedance $Z_0 = 50\Omega$ [5].

We measure the capacitance of 3 types of devices: 20 GHz, 50 GHz, and 100 GHz FSR microresonators with electrodes (cf Figure S7). The decreasing capacitance for higher FSR resonators is in a good agreement with the corresponding electrode length dependency.

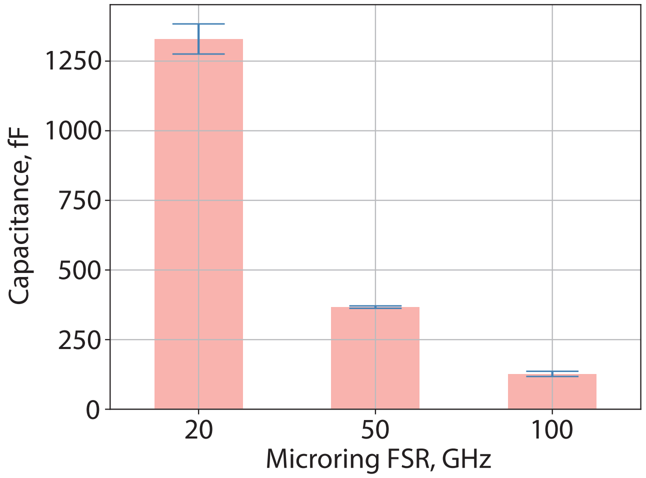

Supplementary Figure 7: Measured capacitance for 3 types of microresonator electrode pairs. The error bars show RMS deviation.

VII. ELECTRO-OPTIC FREQUENCY COMB SIMULATION

In the electro-optic frequency comb experiment presented in Fig 3(c) of the main manuscript we use the RF power of 40 dBm at Ω_{mw}=21 GHz frequency. According to the microwave characterization (see section S4), the electrode capacitance is around 1200 fF. The applied voltage amplitude can be calculated as:

$$V_p = \sqrt{\frac{2P_{\text{mw}}}{C\Omega_{\text{mw}}}}, \tag{5}$$

where $\Omega_{\text{mw}} = 21\text{GHz}$ is the microwave frequency applied. Which gives us $V_p \approx 40\text{V}$.

Now, knowing the linear tuning of the resonator $\partial\nu/\partial V = 41\text{MHz/V}$ (cf section S4) one can calculate the electro-optic coupling rate Γ_{mw} as:

$$\Gamma_{\text{mw}}/2\pi = V_{\text{p}} \times \partial\nu/\partial V \approx 1.5\text{GHz} \tag{6}$$

Full free spectral range resonance shift (21 GHz) would correspond to an intra-cavity electro-optic phase shift of 2π. Therefore the phase modulation amplitude can be estimated as:

$$\beta = \frac{\Gamma_{\text{mw}}}{\text{FSR}} \approx 0.14\pi \tag{7}$$

For the simulations we consider an optical ring coupled to a bus waveguide with external coupling rate $\kappa_{\text{ex}} = 16$ MHz and internal loss rate $\kappa_0 = 65$ MHz. The values are taken from linear optical measurements. The cavity is excited

by a monochromatic laser with photon flux $s_{\text{in}} = P/\hbar\omega_p$ (P is the input power) and frequency ω_p, which is close to resonance frequency ω_0. In the experiment we keep the pump on resonance ($\omega_p = \omega_0$) however some deviations might appear due to the thermal shifts of the resonance under high microwave power. The corresponding linear equation of motion for the slowly varying mode amplitude b_μ can be written as:

$$\partial_t b_\mu + (\frac{\kappa_{\text{ex}}}{2} + \frac{\kappa_0}{2})b_\mu + i(\omega_0 - \omega_p)b_\mu = \sqrt{\kappa_{ex}} s_{\text{in}} \delta_{\mu,0} \tag{8}$$

Under electro-optic modulation, the neighboring cavity modes start to couple with a coupling rate Γ_{mw}. Taking the electro-optic coupling into account, we derive the following coupled mode equation analogically to [6]:

$$\partial_t b_\mu + (\frac{\kappa_{\text{ex}}}{2} + \frac{\kappa_0}{2})b_\mu + i(\omega_0 - \omega_p)b_\mu = \sqrt{\kappa_{ex}} s_{\text{in}} \delta_{\mu,0} + i\frac{\Gamma_{\text{mw}}}{2}(b_{\mu+1} + b_{\mu-1}) \tag{9}$$

As discussed in the main text the group velocity dispersion (D_2) is measured to be small compared to the cavity linewidth over the EO comb spectral range and therefore it can be neglected for simulations. We solve this equation numerically in the time domain using split-step method analogically to the one presented in [7].

VIII. SUPERCONTINUUM AND SECOND-HARMONIC GENERATION IN HYBRID WAVEGUIDES

Finally, we demonstrate supercontinuum generation with combined $\chi^{(2)}$ and $\chi^{(3)}$ nonlinear optical processes using the hybrid waveguides, as shown in Fig. 8(a). A centimeter-long waveguide is pumped with an ultrafast femtosecond pulse laser at 1560 nm center wavelength, with 90 fs pulse duration, 100 MHz repetition rate, and average power up to 100 mW. We observe octave-spanning supercontinuum generation mediated by the $\chi^{(3)}$ nonlinearity, together with simultaneous second harmonic generation (SHG) due to optical field in $LiNbO_3$. As observed already in AlN and LNOI waveguides [8, 9], provided that the field from the supercontinuum overlaps with the SHG signal at $\sim$780 nm, this enables direct measurement of the carrier envelope-offset frequency f_{ceo} at the waveguide output with a photodetector. In Fig. 8(c), we observe isolated SHG beginning with 2 mW average power incident on the waveguide ($\sim$ 0.6 mW in the waveguide accounting insertion loss), while supercontinuum generation is yet to take place. When the incident (on-chip) power increases to 90 mW (28 mW on chip) , we observe spectral broadening towards visible wavelengths. Importantly, a dispersive wave is formed at wavelengths around 710 nm. In this experiment, the insertion loss is higher than the one presented in the main text due to the fact, that coupling is performed using free-space lenses, not lensed fibers. As shown in Fig. 8(d), we obtain the fundamental repetition rate of the supercontinuum as well as the f_{ceo} in the RF spectrum, which shares similar laser noise as the seed mode-locked laser input. We emphasize that, even though the laser pumps the waveguide fundamental mode at 1560 nm, the second harmonic is generated in a higher-order waveguide mode that is phase-matched to the pumped fundamental mode. Nevertheless, it enables direct f_{ceo} measurement with a signal-to-noise ratio of 17 dB within a resolution bandwidth of 30 kHz, sufficient for f_{ceo} stabilization and optical cycle counting. Recent studies [10, 11] have shown that bare Si_3N_4 waveguides can simultaneously exhibit $\chi^{(3)}$ and $\chi^{(2)}$ nonlinearities, and the latter is optically induced by the photogalvanic effect and the formation of self-organized nonlinear grating. However, SHG in these bare Si_3N_4 waveguides suffers from the competition between the $\chi^{(3)}$ and $\chi^{(2)}$ processes, thus suffers from limited power handling capability due to the fact that SHG can be quenched by the simultaneous supercontinuum generation [11]. Our method using hybrid waveguides overcomes this challenge and enables co-generation of SHG and SCG, as the $\chi^{(3)}$ and $\chi^{(2)}$ nonlinearities are inherited from the intrinsic material properties.

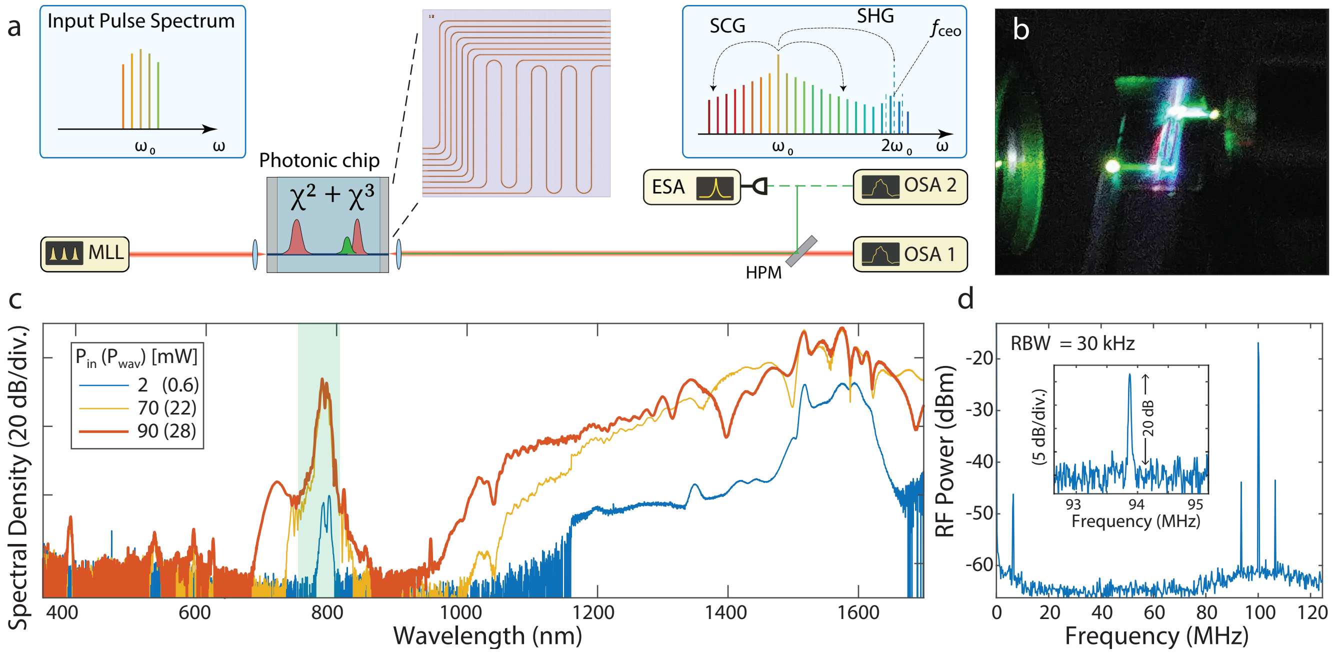

Supplementary Figure 8: Supercontinuum generation and carrier-envelope frequency (f_{ceo}) measurement. (a) Experimental schematic for supercontinuum in a $\chi^{(2)}, \chi^{(3)}$ $LiNbO_3$ waveguide, with input pulse comb spectrum on the left, and a $\chi^{(3)}$-based supercontinuum, overlapped with a $\chi^{(2)}$-based second-harmonic signal, on the right. Chip layout in the centre. Light is coupled in and out of the chip via lenses (5 dB insertion loss). MLL: Mode-locked laser, ESA: Electronic spectrum analyser. OSA: Optical spectrum analyser, HPM: spectral high-pass mirror. (b) Photo of the supercontinuum process in experiment. Blue and Green light corresponds to higher harmonic generation. (c) Total output spectrum for different incident (in waveguide) power levels P_{in} (P_{wav}). (d) f_{ceo} beatnote and $f_{rep} = 100$ MHz repetition rate beatnote, detected at $P_{wav} = 28$ mW.

SUPPLEMENTARY TABLE 1

Reference	Intrinsic Q-factors	Linear optical loss	$V_\pi L$ product (Tuning rate)	Insertion loss	Wafer-level fabrication	Statistical analysis
This work	$4{\cdot}10^6$	0.1 dB/cm	8.8 V·cm (42 MHz·V^{-1})	2.5 dB/facet	Yes	Yes
[12]	10^7	0.027 dB/cm	No data	No data	Yes	No
[13]	No data	No data	13.4 V·cm	6.5 dB/facet	No	No
[14]	$2.5{\cdot}10^6$	No data	(500 MHz·V^{-1})	No data	Yes	No
[15]	No data	$0.2{\pm}0.4$ dB/cm	No data	5 dB/facet	No	No
[16]	$1.8{\cdot}10^6$	0.27 dB/cm	No data	1.7 dB/facet^b	Yes	Yes
[17]	$7.68{\cdot}10^5$	0.2 dB/cm	5.1 V·cm^a	6.5 dB/facet	Yes	No
[18]	No data	No data	6.2 V·cm	No data	Yes	No
[19]	No data	7 dB/cm	6 V·cm	>10 dB/facet	Yes	No

Supplementary Table I: Comparison of the main metrics described in the paper with other related publications on ridge-waveguide and heterogeneously integrated $LiNbO_3$ integrated photonics. The $V_\pi L$ product is given for a single-arm phase modulation. ^{a} The value is estimated from the microresonator tuning rate. ^{b} The insertion loss value is taken from another work published by the same group [20].

REFERENCES

[1] M. H. P. Pfeiffer, C. Herkommer, J. Liu, T. Morais, M. Zervas, M. Geiselmann, and T. J. Kippenberg, IEEE Journal of Selected Topics in Quantum Electronics **24**, 1 (2018).
[2] C. Wang, M. Zhang, X. Chen, M. Bertrand, A. Shams-Ansari, S. Chandrasekhar, P. Winzer, and M. Lončar, Nature **562**, 101 (2018).
[3] M. He, M. Xu, Y. Ren, J. Jian, Z. Ruan, Y. Xu, S. Gao, S. Sun, X. Wen, L. Zhou, L. Liu, C. Guo, H. Chen, S. Yu, L. Liu, and X. Cai, Nature Photonics **13**, 359 (2019), arXiv:1807.10362.
[4] James E. Toney, *Lithium Niobate Photonics* (Artech House Publishers, 2015).
[5] T. Reveyrand, in *2018 International Workshop on Integrated Nonlinear Microwave and Millimetre-wave Circuits (INMMIC)* (IEEE, 2018) pp. 1–3.
[6] A. Dutt, M. Minkov, Q. Lin, L. Yuan, D. A. B. Miller, and S. Fan, Nature Communications **10**, 3122 (2019).
[7] A. K. Tusnin, A. M. Tikan, and T. J. Kippenberg, Physical Review A **102**, 023518 (2020).
[8] D. D. Hickstein, H. Jung, D. R. Carlson, A. Lind, I. Coddington, K. Srinivasan, G. G. Ycas, D. C. Cole, A. Kowligy, C. Fredrick, S. Droste, E. S. Lamb, N. R. Newbury, H. X. Tang, S. A. Diddams, and S. B. Papp, Physical Review Applied **8**, 014025 (2017).
[9] M. Yu, B. Desiatov, Y. Okawachi, A. L. Gaeta, and M. Lončar, Optics Letters **44**, 1222 (2019).
[10] A. Billat, D. Grassani, M. H. P. Pfeiffer, S. Kharitonov, T. J. Kippenberg, and C.-S. Brès, Nature Communications **8**, 1016 (2017).
[11] D. D. Hickstein, D. R. Carlson, H. Mundoor, J. B. Khurgin, K. Srinivasan, D. Westly, A. Kowligy, I. I. Smalyukh, S. A. Diddams, and S. B. Papp, Nature Photonics **13**, 494 (2019).
[12] M. Zhang, C. Wang, R. Cheng, A. Shams-Ansari, and M. Lončar, Optica **4**, 1536 (2017).
[13] N. Boynton, H. Cai, M. Gehl, S. Arterburn, C. Dallo, A. Pomerene, A. Starbuck, D. Hood, D. C. Trotter, T. Friedmann, C. T. DeRose, and A. Lentine, Opt. Express **28**, 1868 (2020).
[14] M. Zhang, C. Wang, Y. Hu, A. Shams-Ansari, T. Ren, S. Fan, and M. Lončar, Nature Photonics **13**, 36 (2019), arXiv:1809.08638.
[15] L. Chang, M. H. P. Pfeiffer, N. Volet, M. Zervas, J. D. Peters, C. L. Manganelli, E. J. Stanton, Y. Li, T. J. Kippenberg, and J. E. Bowers, Opt. Lett. **42**, 803 (2017).
[16] K. Luke, P. Kharel, C. Reimer, L. He, M. Loncar, and M. Zhang, Opt. Express **28**, 24452 (2020).
[17] A. N. R. Ahmed, S. Shi, A. J. Mercante, and D. W. Prather, Opt. Express **27**, 30741 (2019).
[18] A. Rao, A. Patil, P. Rabiei, A. Honardoost, R. DeSalvo, A. Paolella, and S. Fathpour, Opt. Lett. **41**, 5700 (2016).
[19] S. Jin, L. Xu, H. Zhang, and Y. Li, IEEE Photonics Technology Letters **28**, 736 (2016).
[20] L. He, M. Zhang, A. Shams-Ansari, R. Zhu, C. Wang, and L. Marko, Optics Letters **44**, 2314 (2019), arXiv:1902.08969.
